# Supplementary material for: Active site specificity profiling datasets of matrix metalloproteinases (MMPs) 1, 2, 3, 7, 8, 9, 12, 13 and 14
Source: Data Brief. 2016 Feb 22;7:299–310. doi: 10.1016/j.dib.2016.02.036 (PMC4777984; doi:10.1016/j.dib.2016.02.036)
Supplement: Supplementary file 10 — Supplementary material [file mmc10.zip › WebPICS_hMMP8_G_1%/P2prime.html]

 

PICS results


|  |  |
| --- | --- |
| **P2prime\_H**  5 in 120 sites   4.2 %    effects > 10 perc. pnts.  (vice-versa in brackets)  P3\_I: 32.5 (18.0) |  |
| **P2prime\_I**  13 in 120 sites   10.8 %    effects > 10 perc. pnts.  (vice-versa in brackets)  P2\_F: 15.6 (22.5)   P1prime\_V: 13.1 (14.2)   P3prime\_N: 22.5 (29.2) |  |
| **P2prime\_K**  25 in 120 sites   20.8 %    effects > 10 perc. pnts.  (vice-versa in brackets)  P2\_K: 14.8 (33.7)   P1\_A: 17.2 (33.0)   P1\_N: -11.2 (-12.1)   P1prime\_L: 23.2 (11.9)   P3prime\_G: 19.8 (17.1) |  |
| **P2prime\_Q**  12 in 120 sites   10.0 %    effects > 10 perc. pnts.  (vice-versa in brackets)  P3\_H: 20.8 (50.0)   P3\_V: 14.2 (13.1)   P2\_W: 30.0 (90.0)   P1\_P: 25.8 (34.4)   P1prime\_F: 19.2 (32.9)   P3prime\_V: 25.0 (30.0) |  |
| **P2prime\_R**  8 in 120 sites   6.7 %    effects > 10 perc. pnts.  (vice-versa in brackets)  P3\_I: 17.5 (15.5) |  |
| **P2prime\_V**  16 in 120 sites   13.3 %    effects > 10 perc. pnts.  (vice-versa in brackets)  P2\_F: 11.3 (20.0)   P1\_S: 13.3 (15.3) |  |
